# Enhanced Production of Acarbose and Concurrently Reduced Formation of Impurity C by Addition of Validamine in Fermentation of *Actinoplanes utahensis* ZJB-08196

**DOI:** 10.1155/2013/705418

**Published:** 2013-01-22

**Authors:** Ya-Ping Xue, Jun-Wei Qin, Ya-Jun Wang, Yuan-Shan Wang, Yu-Guo Zheng

**Affiliations:** ^1^Institute of Bioengineering, Zhejiang University of Technology, Hangzhou, Zhejiang 310014, China; ^2^Engineering Research Center of Bioconversion and Biopurification of Ministry of Education, Zhejiang University of Technology, Hangzhou, Zhejiang 310014, China; ^3^Huadong Medicine Co., Ltd., Hangzhou, Zhejiang 310011, China

## Abstract

Commercial production of acarbose is exclusively via done microbial fermentation with strains from the genera of *Actinoplanes*. The addition of C_7_N-aminocyclitols for enhanced production of acarbose and concurrently reduced formation of impurity C by cultivation of *A. utahensis* ZJB-08196 in 500-mL shake flasks was investigated, and validamine was found to be the most effective strategy. Under the optimal conditions of validamine addition, acarbose titer was increased from 3560 ± 128 mg/L to 4950 ± 156 mg/L, and impurity C concentration was concurrently decreased from 289 ± 24 mg/L to 107 ± 29 mg/L in batch fermentation after 168 h of cultivation. A further fed-batch experiment coupled with the addition of validamine (20 mg/L) in the fermentation medium prior to inoculation was designed to enhance the production of acarbose. When twice feedings of a mixture of 6 g/L glucose, 14 g/L maltose, and 9 g/L soybean flour were performed at 72 h and 96 h, acarbose titer reached 6606 ± 103 mg/L and impurity C concentration was only 212 ± 12 mg/L at 168 h of cultivation. Acarbose titer and proportion of acarbose/impurity C increased by 85.6% and 152.9% when compared with control experiments. This work demonstrates for the first time that validamine addition is a simple and effective strategy for increasing acarbose production and reducing impurity C formation.

## 1. Introduction

**Scheme 1:**
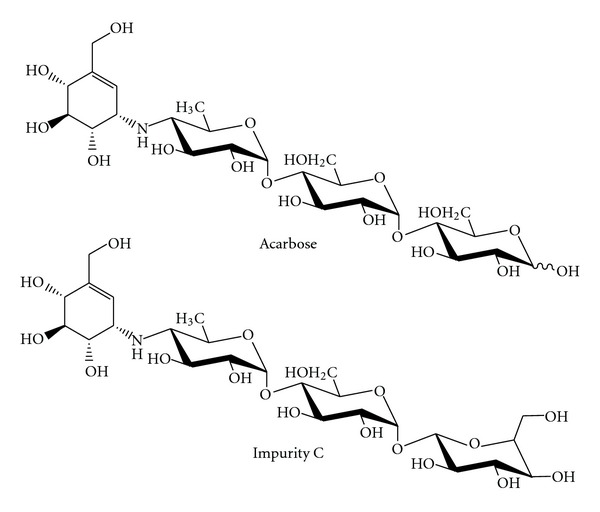
Structures of acarbose and impurity C.

The *α*-glycosidase inhibitor acarbose has been used in many countries in the therapy of diabetes type II, in order to enable patients to better control blood sugar contents while living with starch-containing diets. It is one of the latest successful products of bacterial secondary metabolism to be introduced into the pharmaceutical world market [1–5]. The structure of acarbose consists of an unsaturated cyclitol (valienol), a 4-amino-4,6-dideoxyglucose, and maltose (Scheme 1). The valienol and 4-amino-4,6-dideoxyglucose are linked via an amino bridge mimicking an N-glycosidic bond. This acarviosyl moiety is primarily responsible for the inhibitory effect on *α*-glycosidases [6–8].

So far, commercial production of acarbose is exclusively via microbial fermentation with strains from the genera of *Actinoplanes*. Many experiments including producer strain mutagenesis and screening, media formula, fermentation conditions, and acarbose isolation and purification have been conducted in order to increase acarbose yield because of its high commercial value [1, 9–11]. However, major difficulties still exist in improving the yield of acarbose, leading to a high cost for its manufacture. Recently, Li et al. [12, 13] reported the medium optimization and scale-up strategy for acarbose fermentation by *Actinoplanes* sp. A56 and developed an optimized industrial fermentation processes for acarbose production, as a result about 5000 mg/L of acarbose was obtained. Our group has extensively studied the production of acarbose [14–18]. A high acarbose-producing mutant stain *A. utahensis* ZJB-08196 was isolated by mutagenesis and screening method [14]. Fed-batch fermentation with *A. utahensis* ZJB-08196 at elevated osmolality via intermittently feeding of necessary components regarding acarbose formation afforded a peak acarbose titer of 4878 mg/L [15]. By exogenous addition of *S*-adenosylmethionine (100 *μ*M) after 12 h inoculation in fed-batch fermentation, the maximal titer of acarbose was obtained at 6113 mg/L [19].

Besides acarbose, *Actinoplanes* sp. commonly produces an extensive series of acarviosyl containing acarbose-like components, including impurity A-H [17]. These impurities differ in the number and type of glycosidic bonds which are attached to the acarviosyl core at the reducing and nonreducing ends. Impurity C is identical to that of acarbose except that the *α*-1,4-D-glycosidic linkage of the maltose moiety in acarbose is replaced with a 1,1-linkage in impurity C (Scheme 1) [20]. Due to its high structural similarity to acarbose, impurity C is hard to separate. The purified product still contains some contamination impurity C. Biosynthetic study demonstrated that impurity C was produced in excess during the later fermentation stage of *Actinoplanes*. It can be derived from acarbose. The *α*,*α*-1,4-linkage in the maltose moiety of acarbose can be converted to an *α*,*α*-1,1-linkage in the trehalose moiety of impurity C by glucosyltransferase [21]. Approximately 2300 mg/L of acarbose was produced along with 600 mg/L of impurity C by cultivation of *Actinoplanes *sp. CKD485-16 [20]. *Actinoplanes *sp. A56 produced approximately 5000 mg/L along with 530 mg/L of impurity C at the end of fermentation [13]. A low proportion of acarbose/impurity C makes the purification of acarbose complicated. In order to simplify the separation process, one of the most promising methods is to increase the acarbose production and/or reduce the formation of impurity C by improving the proportion of acarbose/impurity C in the fermentation.

The C_7_N-aminocyclitols are structurally close to sugar moieties, which exhibit glycosidase and glucosyltransferase inhibitory effects [8]. They may play an important role in the metabolic synthesis of acarbose and inhibit the generation of impurity C in fermentation process by *Actinoplanes *sp. In this work, the addition of validamine into the fermentation medium of *A. utahensis* ZJB-08196 was conducted to enhance production of acarbose and concurrently reduce the formation of impurity C in 500-mL shake flasks. The adding concentration and adding time of validamine on acarbose production were optimized. On the basis of above experiments, fed-batch cultivation of *A. utahensis *ZJB-08196 coupled with exogenous addition of validamine in fermentation medium was further investigated. The information obtained in this work would be helpful to industrial production of this important *α*-glycosidase inhibitor, and validamine addition would be a novel and effective strategy for enhancing the acarbose fermentation.

## 2. Materials and Methods

### 2.1. Chemicals

Acarbose standard was purchased from Sigma. Maltose, glucose, sucrose, L-Tyr, monosodium glutamate, glycerol, K_2_HPO_4_·3H_2_O, MgSO_4_·7H_2_O, FeSO_4_·7H_2_O, KCl, FeCl_3_, CaCl_2_, CaCO_3_, and NaOH were of analytical reagent grade purity. Peptone, agar, and soybean meal were obtained from commercial sources. Validamycin A, validamycin B, validamycin D, and validamycin E were separated from crude validamycins by an anion-exchange column packed with resin A600 (Purolite Co. limited, China) according to the method as described previously [22]. Valienamine and validamine were prepared by degradation of validamycin A with *Stenotrophomonas maltophilia *CCTCC M 204024 [23–26]. The purities of these compounds were more than 98%.

### 2.2. Microorganism and Cultivation Conditions

The mutant strain *A. utahensis* ZJB-08196, which was screened by our lab and previously deposited at the China Center for Type Culture Collection (Wuhan, China) as CCTCC M 209022, was used for acarbose production [14]. This strain was stored as spore/mycelial suspensions in 15% (v/v) glycerol solution at −70°C until inoculation. It was spread over agar plates containing (g/L) sucrose 30.0, peptone 2.0, L-Tyr 1.0, K_2_HPO_4_·3H_2_O 1.0, KCl 0.5, MgSO_4_·7H_2_O 0.5, FeSO_4_ 0.1, and agar 20.0, and the initial pH value was adjusted to 7.0. For inoculum, a colony of about 1 × 1 cm^2^ size from a freshly prepared agar plate was inoculated into a 500-mL shake flask containing 100 mL of seed medium and cultivated at 28°C and 200 rpm for 72 h. The seed medium consisted of (g/L) corn starch 15, soybean flour 40, glycerol 20, CaCO_3_ 2.0, and K_2_HPO_4_ 0.5, with pH unadjusted. Batch and fed-batch fermentations were carried out by inoculating 10% (v/v) of the seed culture into 500-mL shake flasks containing 50 mL of fermentation medium at 28°C and 200 rpm. The basal medium for acarbose fermentation consisted of (per L) maltose syrup (containing 11 g/L glucose and 160 g/L maltose) 270 mL, glucose 40.0 g, soybean flour 17.0 g, sodium glutamate 5.0 g, CaCO_3_ 2.5 g, CaCl_2_ 2.5 g, FeCl_3_ 0.5 g, K_2_HPO_4_ 1.0 g, and glycerol 5.0 g, and the initial pH value was adjusted to 7.0.

### 2.3. Effect of C_7_N-Aminocyclitols Addition on Acarbose Fermentation

Sterile-filtered aqueous solutions of C_7_N-aminocyclitols (validamycin A, validamycin B, validamycin D, validamycin E, valienamine, and validamine) were added into the fermentation medium, respectively, to investigate the effect of different C_7_N-aminocyclitols addition on acarbose fermentation. The adding concentration and adding time of validamine on acarbose production were optimized. On the basis of above experiments, an optimal feeding strategy, coupled with exogenous validamine addition (20 mg/L), was performed for maximum acarbose production.

### 2.4. Analytical Methods

Samples (5 mL) were centrifuged at 9,000 ×g for 15 min. The cell precipitates were dried at 80°C to a constant weight for determining dry cell weight (DCW) as the biomass. Acarbose titer and impurity C concentration in the supernatant were analyzed by using a Shimadzu CT0-10ASVP HPLC system according to the method as described previously [14]. Maltose was measured according to known methodologies [27]. Glucose in broth was measured with SBA-40E biosensor (Biology Institute of Shandong Academy of Sciences, China) after dilution (10–100X) with deionized water. Medium osmolality was assayed using an Osmometer, Model 3250, (Advanced, USA) in accordance with the manual.

### 2.5. Statistical Analysis

Each run of fermentation experiments was duplicated three times to ensure reliability and accuracy. Data are the average of three independent sample measurements. The error bars indicate the standard deviation (SD) from the mean of triplicates. Data were analyzed with Student's *t*-test. The difference between contrasting treatments was considered significant when *P* is <0.05 in a two-tail analysis. 

## 3. Results and Discussion

### 3.1. Time Courses of Acarbose Fermentation in Batch Culture of *A. utahensis* ZJB-08196

**Figure 1:**
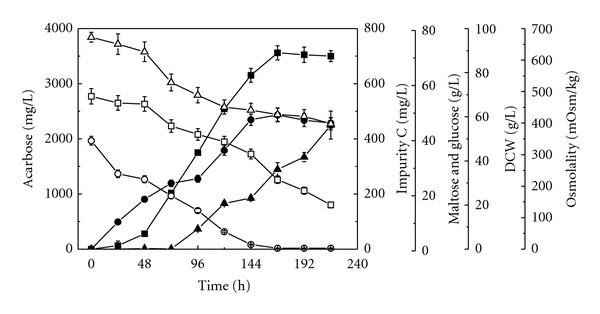
Time course of acarbose accumulation, maltose and glucose consumption, cell growth, and impurity C formation during batch cultivation of *A. utahensis* ZJB-08196 at 28°C and 200 rpm for 216 h in fermentation medium. Symbols: ■: acarbose; ▲: impurity C; •: DCW; □: maltose; ○: glucose; △: osmolality.

The time courses for acarbose accumulation, substrate consumption, cell growth, and impurity C formation in batch culture of *A. utahensis *ZJB-08196 were firstly investigated. Cultivations were performed in 500-mL shake flasks containing 50 mL of fermentation medium at 28°C and 200 rpm, and lasted for 216 h. The results were shown in Figure 1. Glucose, as the most easily utilizable carbon source, was almost exhausted in 144 h. Maltose in the fermentation medium was used as an energy source and as a precursor for acarbose. It was found that maltose was consumed slowly in the early stage of fermentation. After glucose was exhausted, maltose was consumed dramatically, accompanied by the rapid accumulation of acarbose. It was utilized not only as the carbon source for cell growth because of the exhaustion of glucose, but also as the direct precursor for the biosynthesis of acarbose. The pH value was observed to be increased slightly throughout the fermentation process (from 7 to 8). The medium osmolality decreased from 680 mOsm/kg to 389 mOsm/kg along with the consumption of substrate. Biomass increased for up to 6 days. Acarbose formation began at the early growth stages, as a mixed-growth-associated type of secondary metabolite in *A. utahensis* ZJB-08196, different from *Actinoplanes* sp. CKD485-16 [14, 20]. After 168 h of cultivation, acarbose titer reached the maximum value of 3560 ± 128 mg/L. Impurity C, as a byproduct of the metabolism, began to accumulate from 72 h of cultivation with a remarkably increase after 144 h. It is from 168 h that the concentration of impurity C increased continuously along with the decrease of acarbose. It may be explained that an intracellular or cell-bound glucosyltransferase in *A. utahensis* ZJB-08196 converts the *α*-1,4-glycosidic bond to *α*-1,1 of the reducing end glucose moiety of acarbose. At the end of cultivation (216 h), the impurity C concentration reached approximately 450 ± 51 mg/L. According to the fermentation profiles, when the acarbose reached its highest titer (at 168 h), the concentration of impurity C was 289 ± 24 mg/L. It can be calculated that the proportion of acarbose/impurity C was only 12.32. As a byproduct, impurity C seems to be unavoidable. However, the higher concentration of impurity C produced in the fermentation added much pressure to downstream processing due to impurity C structural resemblance to acarbose. The US Food and Drug Administration and other regulatory agencies set strict limits on the content of impurity C (<1.5 percent). To satisfy the mandatory requirements, the purification methods utilize complex process in which a step using a combination of the cation exchanger and the anion exchanger is repeatedly performed and a chromatographic step using the cation exchanger is further conducted [28–30]. The higher proportion of acarbose/impurity C in fermentation would not only make the acarbose purification become simple, but also increased the product yield. It is very necessary to develop strategies to increase acarbose titer and/or decrease impurity C concentration to facilitate the downstream process by improving proportion of acarbose/impurity C. 

### 3.2. Effect of Addition of C_7_N-Aminocyclitols on Acarbose Fermentation in Batch Culture

**Figure 2:**
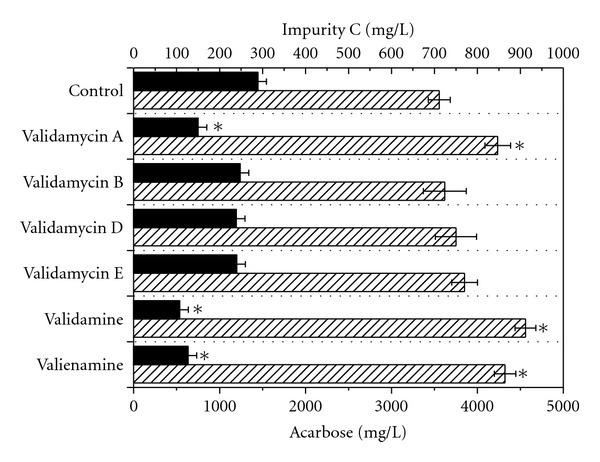
Effect of exogenous addition of C_7_N-aminocyclitols on the acarbose fermentation in batch culture by *A. utahensis *ZJB-08196. Symbols: black bar: impurity C; lined bar: acarbose. Fermentations were carried out at 28°C and 200 rpm for 168 h in fermentation medium with the addition of different C_7_N-aminocyclitols at a final concentration of 80 mg/L prior to the inoculation. The error bars in the figure indicate the standard deviations from three independent samples. *indicates statistic significance (*P* < 0.05) compared to control experiments without the addition of C_7_N-aminocyclitols.

The C_7_N-aminocyclitols exhibited physiological roles in almost all aspects of microorganisms due to their glycosidase and glucosyltransferase inhibitory activity [8, 21]. They may affect the cell growth and metabolite accumulation in microorganism cultures. The spectrum of their biological activities is apparently broader than that was originally thought [8]. Our group has developed a method for the production of validamycin A, validamycin B, validamycin D, and validamycin E with anion-exchange resin A600 and reported the protocol to prepare valienamine and validamine from validamycin A [22–26]. In this work, the effect of addition of C_7_N-aminocyclitols on acarbose fermentation was investigated. Validamycin A, validamycin B, validamycin D, validamycin E, valienamine, and validamine were added to the fermentation medium of *A. utahensis* ZJB-08196 at a final concentration of 80 mg/L prior to inoculation, respectively. The fermentations were carried out for 168 h in 500-mL shake flasks with 50 mL of fermentation medium at 28°C. The results were shown in Figure 2. No significant difference on acarbose titer and impurity C concentration was observed when validamycin B, validamycin D, and validamycin E were added to fermentation medium. Validamycin A, validamine, and valienamine can stimulate acarbose production and inhibit the impurity C formation. Biomass slightly increased after 168 h of cultivation (data no shown). These compounds might be involved with biosynthetic pathways of acarbose as activators and act as the inhibitor of glucosyltransferase. The highest acarbose titer of 4559 ± 121 mg/L and lowest impurity C concentration of 101 ± 23 mg/L were obtained in the presence of validamine. Acarbose titer increased by 28.1%, and impurity C decreased by 65.7% compared with the levels in the absence of validamine. The proportion of acarbose/impurity C was improved from 12.32 to 45.14. The results indicate that validamine can be effectively used for enhanced production of acarbose and concurrently reduced formation of impurity C by cultivation of *A. utahensis *ZJB-08196. In the following experiments, the addition conditions were investigated about the positive effect of validamine on acarbose fermentation.

### 3.3. Effect of Adding Concentration of Validamine on Acarbose Fermentation in Batch Culture

**Figure 3:**
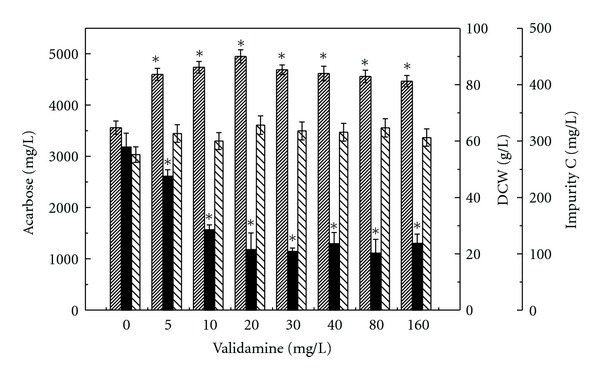
Effect of different concentrations of validamine added on the acarbose fermentation in batch culture by *A. utahensis *ZJB-08196. Symbols: shaded line bar:acarbose; black bar: impurity C; lined bar: DCW. Fermentations were carried out at 28°C and 200 rpm for 168 h in fermentation medium with the exogenous addition of validamine in the range from 0 to 160 mg/L prior to inoculation. The error bars in the figure indicate the standard deviations from three independent samples. *indicates statistic significance (*P* < 0.05) compared to control experiments without the addition of validamine.

Effect of different concentrations of validamine added in the fermentation medium on the formation of acarbose and impurity C was evaluated. Fermentations were carried out at 28°C and 200 rpm for 168 h with the exogenous addition of validamine in fermentation medium in the range from 0 to 160 mg/L prior to inoculation. The results were shown in Figure 3. DCW slightly increased by the addition of validamine. The acarbose titer increased with the increase of validamine concentration from 0 to 20 mg/L. The maximum acarbose titer of 4950 ± 156 mg/L was obtained at 20 mg/L of validamine. Further increase of validamine amount led to a slight drop. The impurity C concentration decreased from 289 ± 24 mg/L to 107 ± 29 mg/L with an increase of validamine concentration from 0 to 20 mg/L. Impurity C reduced by about 62.9% at 20 mg/L of validamine compared with control experiment without addition of validamine. Further increase of validamine amount could not improve acarbose titer. No significant reduction in impurity C formation was found when validamine concentration was beyond 20 mg/L. 

### 3.4. Effect of Adding Time of Validamine on Acarbose Fermentation in Batch Culture

**Figure 4:**
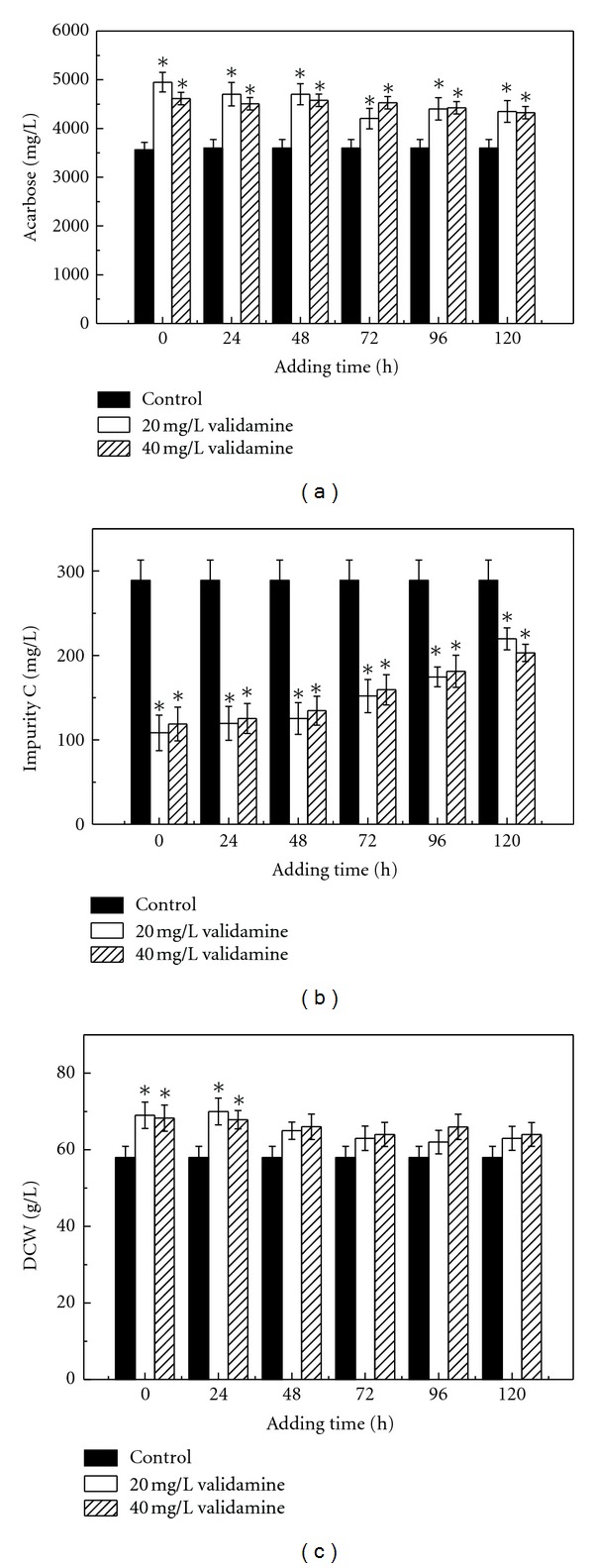
Effect of adding time of validamine on the acarbose production (a), impurity C formation (b), and cells growth (c) in batch culture by *A. utahensis *ZJB-08196. Fermentation was carried out at 28°C and 200 rpm for 168 h in fermentation medium with the addition of validamine at a final concentration of 20 mg/L and 40 mg/L at a different cultivation time. The error bars in the figure indicate the standard deviations from three independent samples. *indicates statistic significance (*P* < 0.05) compared to control experiments without the addition of validamine.

Validamine was added at 0 h, 24 h, 48 h, 72 h, 96 h, and 120 h at final concentrations of 20 mg/L and 40 mg/L to study the effect of its adding time on acarbose fermentation. The results were shown in Figure 4. The addition time of validamine affected biomass. The biomass was increased by about 14% when validamine was added within 24 h after inoculation. The addition of validamine at 20 mg/L or 40 mg/L at all the fermentation time in the range from 0 to 120 h can not only promote acarbose yield, but also reduce the formation of impurity C. About 23.6–39.0% increase in acarbose titer and 12.4–62.9% reduction in impurity C concentration were obtained as compared with control experiments. The addition of validamine at the beginning of the fermentation was beneficial for the improvement of acarbose production and inhibition of impurity C formation. This finding indicated that the response of acarbose biosynthesis to the addition time was closely related to the cellular physiological state of *A. utahensis* ZJB-08196.

**Figure 5:**
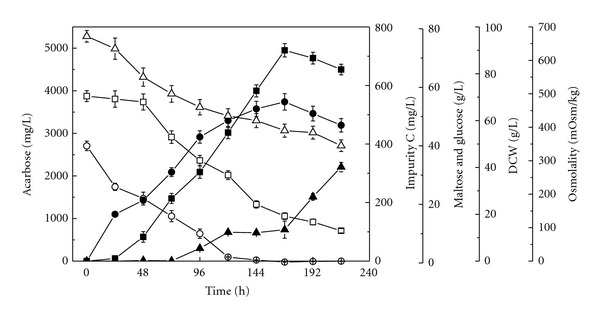
Time courses of acarbose accumulation, maltose and glucose consumption, cell growth and impurity C formation during batch cultivation of *A. utahensis *ZJB-08196 with the addition of validamine at a final concentration of 20 mg/L in fermentation medium prior to inoculation. Symbols: ■: acarbose; ▲: impurity C; •: DCW; □: maltose; ○: glucose; △: osmolality. Fermentations were carried out at 28°C and 200 rpm for 216 h.

Time courses of acarbose formation, maltose and glucose consumption, cell growth, and impurity C accumulation during batch cultivation of *A. utahensis *ZJB-08196 in fermentation medium with the addition of validamine at 20 mg/L at the beginning of fermentation were shown in Figure 5. Acarbose titer reached the maximum value of 4950 ± 56 mg/L at 168 h of cultivation. Impurity C began to accumulate after 72 h and reached 108 ± 12 mg/L at 168 h of cultivation. Its concentration remarkably increased after 168 h and increased continuously along with the decrease of acarbose. Therefore, the optimum fermentation period was 168 h. The medium osmolality decreased from 680 mOsm/kg to 345 mOsm/kg. The result indicated that the addition of validamine will led to a lower osmolality in the late stage of fermentation compared with the experiments without the addition of validamine (398 mOsm/kg). Our previous work revealed that *A. utahensis *ZJB-08196 had a broad osmolality optimum between 309 mOsm/kg and 719 mOsm/kg [15], which is quite different from those of *Actinoplanes* sp. SE50/110 [10] and *Actinoplanes* sp. CKD485-16 [20]. Therefore, the slight decrease of osmolality due to the addition of validamine in the fermentation of *A. utahensis* ZJB-08196 may have no effect on the acarbose production and impurity C formation. 

### 3.5. Effect of Addition of Validamine on the Acarbose Fermentation in Fed-Batch Culture

**Figure 6:**
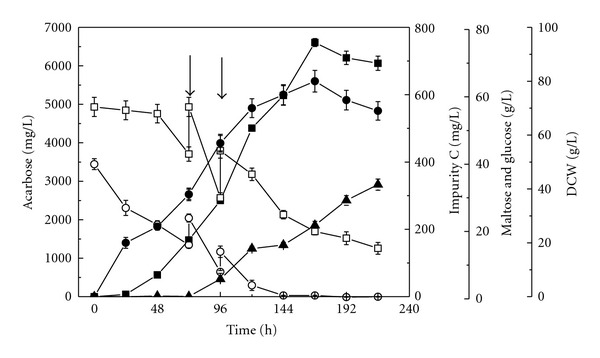
Time courses of acarbose accumulation, maltose and glucose consumption, cell growth, and impurity C formation during fed-batch cultivation of *A. utahensis *ZJB-08196 with the addition of validamine at a final concentration of 20 mg/L in fermentation medium prior to inoculation. Symbols: ■: acarbose; ▲: impurity C; •: DCW; □: maltose; ○: glucose. Fermentations were carried at 28°C and 200 rpm for 216 h. Twice feedings indicated by the arrows; feed volume 5 mL; feed composition: 14.0 g/L maltose, 6.0 g/L glucose, and 9.0 g/L soybean meal.

Fed-batch culture is a common strategy in fermentation process to achieve good production [31]. Effect of addition of validamine on the acarbose fermentation in fed-batch culture was also investigated. Fed-batch fermentations were carried at 28°C and 200 rpm for 216 h with the addition of validamine at a final concentration of 20 mg/L prior to inoculation. A proper feeding mode with the reasonable component constitution was developed for the intermittent fed-batch fermentation process. In our previous work, effect of each component in the feed of basal fermentation medium has been evaluated to examine what components are necessary for acarbose synthesis. The results showed that maltose, glucose, and soybean meal are essential feed components. Feeding of a mixture of 6 g/L glucose, 14 g/L maltose, and 9 g/L soybean flour is suitable for acarbose biosynthesis [15]. We adopted the same feed composition in this work. Considering the acarbose and impurity C yield, as well as the length of fermentation period, effect of feeding time on the biosynthesis of acarbose and impurity C was examined at an interval of 24 h after inoculation. The maximum acarbose titer was obtained when twice feedings of a mixture (5 mL) of 6 g/L glucose, 14 g/L maltose, and 9 g/L soybean flour was performed at 72 h and 96 h. The time course of fermentation was shown in Figure 6. The validamine addition at the beginning of cultivation in fed-batch fermentation by *A. utahensis *ZJB-08196 accelerated the accumulation of acarbose greatly. A maximal acarbose titer of 6606 ± 103 mg/L was obtained at 168 h, which is increased by more than 33.4% compared to the batch culture. Impurity C began to accumulate after 72 h and reached 212 ± 12 mg/L at 168 h of cultivation.

The comparison of the kinetic parameters of acarbose fermentation with different methods is shown in Table 1. The results showed that the addition of validamine played a very important role in *A. utahensis *ZJB-08196 fermentation. On one hand, the addition of validamine in the fermentation broth promoted the consumption rates of glucose and maltose. The specific consumption rates of glucose and maltose increased by 4.4% and 15.1% in batch fermentation compared with the control experiments in the absence of validamine. In fed-batch fermentation, the specific consumption rates of glucose and maltose increased by 47.8% and 71.5%. On the other hand, the addition of validamine in the fermentation increased the maximum acarbose titer, acabose productivity, and proportion of acarbose/impurity C greatly. Compared with control experiments, the maximum acarbose titer, acarbose productivity, and proportion of acarbose/impurity C increased by 39.0%, 39.0%, and 275.5% in batch fermentations, respectively. As for fed-batch fermentations, the maximum acarbose titer, acarbose productivity, and proportion of acarbose/impurity C increased by 85.6%, 85.6%, and 152.9%, respectively. 

The regulative mechanism about validamine on acarbose production and impurity C formation by *A*. *utahensis* ZJB-08196 may be explained from two respects. On one hand, validamine is a glucosyltransferase inhibitor, which can inhibit the *α*,*α*-1,4-linkage in the maltose moiety of acarbose to convert to an *α*,*α*-1,1-linkage in the trehalose moiety of impurity C, thus reducing the formation of impurity C. On the other hand, validamine is involved with biosynthetic pathway of the acarbose as an activator. In order to discover the real mechanism of the effects of validamine on acarbose production and impurity C formation by *A. utahensis *ZJB-08196, we are working on the analysis of the transcription level of structural genes and global regulatory genes of acarbose and impurity C with the addition of validamine in fermentation medium by the cultivation of* A. utahensis *ZJB-08196.

The prevalence of diabetes, especially type II diabetes mellitus, is markedly increasing throughout the world [32]. Facing such an increasingly worsening trend, the research and production of diabetes drugs including acarbose are becoming more and more critical to reduce the healthcare spending [33]. Commercial production of validamine can be performed by microbial degradation of validamycin in one step fermentation with high yield [25]. Validamycin is one of the largest yield antibiotics. Its fermentation concentration reaches 30,000 *μ*g/mL broth, and its cost is very low. This shows that validamine can be obtained with cheap price. In addition, the amount of validamine added to the fermentation medium is very low (20 mg/L). Therefore, the method of using validamine to enhance the production of acarbose and concurrently reduce the formation of impurity C is potential and hopeful in industrial application.

## 4. Conclusions

Acarbose, one of the most popular pharmaceuticals in the therapy of diabetes type II, is primarily manufactured using *Actinoplanes* sp. in a fermentation process. A higher acarbose titer and proportion of acarbose/impurity C in fermentation broth would facilitate the downstream process. The current study reveals for the first time that the addition of validamine is useful for the enhanced production of acarbose and concurrently reduced formation of impurity C by the cultivation of *A. utahensis *ZJB-08196, which would bring more convenience to product separation. Remarkable improvements on acarbose titer, acarbose productivity, and proportion of acarbose/impurity C were successfully achieved when 20 mg/L of validamine was added in the fermentation medium prior to inoculation both in batch and fed-batch cultures. The acarbose titer and acarbose productivity obtained in fed-batch culture in this work are the highest that have been reported for all the submerged fermentations of *A. utahensis* [10, 12–15, 19, 20, 27]. This simple and effective strategy would be of great value to acarbose production on commercial scale.

## Figures and Tables

**Table 1:** The kinetic parameters of acarbose fermentation with different fermentation methods.

Parameter	Cultivation method		
	Control	A	B
Maximum acarbose titer (mg/L)	3560	4950	6606
Specific growth rate (1/h)	0.0086	0.0094	0.010
Acarbose productivity (mg/L/h)	21.19	29.46	39.32
Specific maltose consumption rate (1/h)	0.0053	0.0061	0.0103
Specific glucose consumption rate (1/h)	0.0046	0.0048	0.0068
Impurity C concentration (mg/L)	289	107	212
Impurity C productivity (mg/L/h)^a^	1.49	0.64	1.26
Acarbose/impurity C^a^	12.32	46.26	31.16

^a^The value was calculated when acarbose reached its highest titer in the fermentation.

Method A: validamine was added at a final concentration of 20 mg/L in the fermentation medium prior to inoculation in batch culture.

Method B: validamine was added at a final concentration of 20 mg/L in the fermentation medium prior to inoculation in fed-batch culture. Twice feedings of a mixture of 6 g/L glucose, 14 g/L maltose, and 9 g/L soybean flour were performed at 72 h and 96 h.

## References

[1] Wehmeier UF, Piepersberg W (2004). Biotechnology and molecular biology of the *α*-glucosidase inhibitor acarbose. *Applied Microbiology and Biotechnology*.

[2] Laube H (2002). Acarbose: an update of its therapeutic use in diabetes treatment. *Clinical Drug Investigation*.

[3] Balfour JA, McTavish D (1993). Acarbose. An update of its pharmacology and therapeutic use in diabetes mellitus. *Drugs*.

[4] Chiasson JL, Josse RG, Gomis R, Hanefeld M, Karasik A, Laakso M (2002). Acarbose for prevention of type 2 diabetes mellitus: the STOP-NIDDM randomised trial. *The Lancet*.

[5] Delorme S, Chiasson JL (2005). Acarbose in the prevention of cardiovascular disease in subjects with impaired glucose tolerance and type 2 diabetes mellitus. *Current Opinion in Pharmacology*.

[6] Zhang CS, Stratmann A, Block O (2002). Biosynthesis of the C_7_-cyclitol moiety of acarbose in *Actinoplanes *species SE50/110. 7-O-phosphorylation of the initial cyclitol precursor leads to proposal of a new biosynthetic pathway. *Journal of Biological Chemistry*.

[7] Mahmud T, Tornus I, Egelkrout E (1999). Biosynthetic studies on the *α*-glucosidase inhibitor acarbose in *Actinoplanes* sp.: 2-epi-5-epi-valiolone is the direct precursor of the valienamine moiety. *Journal of the American Chemical Society*.

[8] Mahmud T (2003). The C_7_N aminocyclitol family of natural products. *Natural Product Reports*.

[9] Rodriguez JF, De Lucas A, Carmona M, Cañas F (2008). Application of ion exchange to purify acarbose from fermentation broths. *Biochemical Engineering Journal*.

[10] Beunink J, Schedel M, Steiner U Osmotically controlled fermentation process for the preparation of acarbose.

[11] Wei SM, Chen G, Tian W, Liu DS, Zhang YX, He JY (2008). The feeding techniques in acarbose fermentation. *Chinese Journal of New Drugs*.

[12] Li KT, Wie SJ, Huang L, Cheng X (2012). An effective and simplified scale-up strategy for acarbose fermentation based on the carbon source control. *World Journal of Microbiology and Biotechnology*.

[13] Li KT, Zhou J, Wei SJ, Cheng X (2012). An optimized industrial fermentation processes for acarbose production by *Actinoplanes* sp. A56. *Bioresource Technology*.

[14] Wang YJ, Liu LL, Feng ZH, Liu ZQ, Zheng YG (2011). Optimization of media composition and culture conditions for acarbose production by *Actinoplanes utahensis* ZJB-08196. *World Journal of Microbiology & Biotechnology*.

[15] Wang YJ, Liu LL, Wang YS, Xue YP, Zheng YG, Shen YC (2012). Actinoplanes utahensis ZJB-08196 fed-batch fermentation at elevated osmolality for enhancing acarbose production. *Bioresource Technology*.

[16] Feng ZH, Wang YS, Zheng YG (2011). A new microtiter plate-based screening method for microorganisms producing *α*-amylase inhibitors. *Biotechnology and Bioprocess Engineering*.

[17] Wang YJ, Zheng YG, Xue YP, Wang YS, Shen YC (2011). Analysis and determination of anti-diabetes drug acarbose and its structural analogs. *Current Pharmaceutical Analysis*.

[18] Wang YJ, Dong FZ, Yu L, Zheng YG (2012). Study on acarbose adsorption performance of cation exchanger SAC 001×7. *Journal of Chemical Engineering of Chinese Universities*.

[19] Sun LH, Li MG, Wang YS, Zheng YG (2012). Significantly enhanced production of acarbose in fed-batch fermentation with the addition of S-adenosylmethionine. *Journal of Microbiology and Biotechnology*.

[20] Choi BT, Shin CS (2003). Reduced formation of byproduct component C in acarbose fermentation by *Actinoplanes* sp. CKD485-16. *Biotechnology Progress*.

[21] Choi BT, Shin CS (2004). Isolation and characterization of a novel intracellular glucosyltransferase from the acarbose producer *Actinoplanes* sp. CKD485-16. *Applied Microbiology and Biotechnology*.

[22] Zheng YG, Xue YP, Shen YC, Wu YF (2006). Separation and preparation of validamycin A and validamycin B using anion-exchange resin. *Chemical Engineering Communications*.

[23] Zheng YG, Xue YP, Shen YC (2006). Production of valienamine by a newly isolated strain: *Stenotrophomonas maltrophilia*. *Enzyme and Microbial Technology*.

[24] Xue YP, Zheng YG, Shen YC (2007). Enhanced production of valienamine by *Stenotrophomonas maltrophilia* with fed-batch culture in a stirred tank bioreactor. *Process Biochemistry*.

[25] Zheng YG, Chen XL, Xue YP, Wang YS, Shen YC Microbe method for producing valienamine and validamine.

[26] Xue YP, Zheng YG, Chen XL, Shen YC (2007). Quantitative determination of valienamine and validamine by thin-layer chromatography. *Journal of Chromatographic Science*.

[27] Jiang W, Yang YT, Cai YM, Guo MJ, Chu J (2010). Comprehensive effects of maltose concentration and medium osmotic pressure on acarbose in *Actinoplanes* sp. fermentation. *Chinese Journal of Pharmaceuticals*.

[28] Mihaljevic K, Azaric J, Bajic B, Mrsa V, Kokanj D Acarbose purification process.

[29] Lin CL, Huang TL, Chen JK, Wu CS Purification process for manufacturing a high pure acarbose.

[30] Keri V, Deak L, Szabo C Purification of acarbose by chromatography with non-aromatic, strong acid cation exchanger.

[31] Ubiyvovk VM, Ananin VM, Malyshev AY, Kang HA, Sibirny AA (2011). Optimization of glutathione production in batch and fed-batch cultures by the wild-type and recombinant strains of the methylotrophic yeast *Hansenula polymorpha* DL-1. *BMC Biotechnology*.

[32] Ning G, Hong J, Bi Y (2009). Progress in diabetes research in China. *Journal of Diabetes*.

[33] Wang W, McGreevey WP, Fu C (2009). Type 2 diabetes mellitus in China: a preventable economic burden. *American Journal of Managed Care*.

